# Interaction Network Between Frugivorous Birds and Plants in Karst Habitats

**DOI:** 10.1002/ece3.72087

**Published:** 2025-09-17

**Authors:** Guohai Wang, Yanru Wang, Can Zhou, Huangmin Zhang, Lijuan Wei, Dengpan Nong, Chi Yung Jim, Qihai Zhou

**Affiliations:** ^1^ College of Agriculture and Biology Guangxi Minzu Normal University Chongzuo China; ^2^ Key Laboratory of Ecology of Rare and Endangered Species and Environmental Protection, Ministry of Education Guilin China; ^3^ The Chongzuo White‐Headed Langur Field Observation and Research Station of Guangxi Chongzuo China; ^4^ College of Mathematics and Computer Science Guangxi Minzu Normal University Chongzuo China; ^5^ Department of Social Sciences and Policy Studies Education University of Hong Kong Hong Kong China

**Keywords:** bird and fruit trait, frugivorous bird, fruiting plant, functional role, interaction network, karst habitat

## Abstract

The karst region of southwest China is characterized by fragmented habitats featuring high plant diversity but low biological resource availability. Furthermore, high‐intensity human activities degrade natural vegetation, reduce landscape connectivity, and alter the bird–plant interaction network. To characterize plant–frugivorous interaction networks in karst habitats, we established six 3 to 5 km transects within Guangxi's Chongzuo White‐headed Langur National Nature Reserve, conducting year‐round observations of 18 frugivorous birds foraging on 14 fruiting plant species. We calculated four network‐level metrics (characterizing network structure) and three species‐level metrics (evaluating bird and plant contributions to network structure). Then, we analyzed how species traits influenced their network roles. Compared with the networks (*N* = 1000) generated by the null model, the observed network exhibited lower connectance (C_*z*‐score_ = −24.86), higher weighted nestedness (*w*NODF_*z*‐score_ = −1.34), higher specialization (*H*_2_ *´*_*z*‐score_ = 75.39), and higher modularity (*Q*_*z*‐score_ = 35.85). Rainy seasons showed higher *z*‐scores for these metrics than dry seasons. Under three extinction scenarios, bird robustness ranged from 0.459 to 0.936, while plant robustness ranged from 0.404 to 0.872. Network stability was compromised when bird or plant species with the highest centrality values were removed, with plant removal being more detrimental. *Pycnonotus jocosus* , *P. aurigaster* , and *P. sinensis* showed the highest species degree and species strength among birds. Meanwhile, *Ficus altissima* , *F. concinna* , and *Camphora officinarum* exhibited the highest values among plants. Bird body mass correlated negatively with species degree and species strength but positively with specialization. Body length correlated positively with species degree and negatively with specialization. The fruit abundance index (FAI) correlated positively with plant species degree and species strength. Our findings highlighted the seasonal differences in the frugivorous bird–plant network in karst habitats and the species traits affecting network functional roles. These results enrich the theoretical framework of the plant–frugivorous interaction network in karst ecosystems, providing a foundation for further analysis of the impact of karst habitat fragmentation on network structure.

## Graphical abstract

The results suggested that interaction networks in a nature reserve were temporally complex and variable. A network approach could offer an important monitoring tool for detecting crucial ecosystem functions of bird–plant associations.

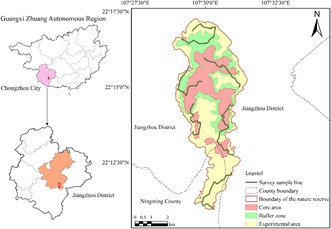

## 1 Introduction

The interactions between plants and frugivorous birds constitute an important ecosystem service (Vizentin‐Bugoni et al. 2019). They contribute to the survival of seed dispersers and the balanced distribution of propagules for many plant communities (González‐Castro et al. 2015; Schupp et al. 2010). Frugivorous birds are primary seed dispersers for fleshy‐fruited plants (Zhang et al. 2022). They eject undamaged seeds from their digestive tract or regurgitate them in habitats suitable for germination (Dehling et al. 2014). Furthermore, frugivorous birds vary in abundance, mobility, species diversity, and morphological traits (Jordano et al. 2011; Rumeu et al. 2023). Concurrent foraging of different fruit types by these birds can enhance the ecological functional diversity of interaction networks (Saavedra et al. 2014). Unraveling the structure and dynamics of such mutualistic connections can enhance understanding of the intricacies of ecological communities, given their paramount role in shaping evolution, biodiversity distribution patterns, and ecosystem service functioning (Guimarães 2020).

Interaction networks of plant‐frugivorous birds are complex and diverse, as some birds can interact with a variety of plants (Angulo‐Ortiz et al. 2024), while others interact with only a few (Zhang et al. 2022). These intricate interactions can be assessed by network analysis to calculate relevant topological metrics such as connectance (which describes the quantity of interactions), robustness (which refers to the tolerance of communities to species extinctions), and modularity, nestedness, and specialization (Li et al. 2022; Howes et al. 2023). Evaluating dynamic parameter changes can disclose the ecological driving factors regulating the interaction networks (Saavedra et al. 2011; Donoso et al. 2017). For instance, habitat fragmentation can alter the network structure by changing species richness and the functional composition of communities. These changes include behavioral responses such as partner switches, which can reduce interaction nestedness and functional trait diversity (Banks‐Leite et al. 2012; Cazetta and Fahrig 2022). When interactions are highly specialized, specialist frugivorous cannot be replaced by others. Thus, landscape change may cause interaction loss and impair plant seed dispersal capacity (Quitián et al. 2018; Montoya‐Arango et al. 2019).

The trait matching between frugivorous birds and plants is an important driver shaping the functional role of species in interaction networks (Teodosio‐Faustino et al. 2021). For example, the nutritional composition, size, availability, and color of fruits affect the behavioral decisions of frugivorous birds (Blendinger et al. 2015; Ordano et al. 2017). Bird species with larger morphology tend to interact with more plant species and occupy a more central role in networks. Bird richness also strongly influences interaction frequency and habitat selection (Dehling et al. 2016). Therefore, species with distinct traits perform more unique functional roles in maintaining the robustness of ecological community structure (Ruggera et al. 2016; Moulatlet et al. 2023). Considering the importance of frugivory interactions in ecosystem restoration, it is necessary to identify the species that play important functional roles in maintaining the structure of the interaction networks and evaluate whether they have particular ecological and morphological traits (Acevedo‐Quintero et al. 2020).

The karst region of southwest China represents a unique geological‐ecological landscape characterized by highly exposed rocks, limited surface water, and sparsely and unevenly distributed soils (Nie et al. 2014). The soils form disparate and fragmented patches of varying sizes in the landscape, influencing the diversity and spatial distribution patterns of fruiting plants (Liu et al. 2022). Furthermore, human activities such as resource extraction, arable farming, urban expansion, and road construction have continually decreased natural vegetation cover and exacerbated habitat fragmentation (Yuan and Li 2023). Such impacts degrade the landscape continuity between patches, hindering bird movement and ultimately affecting the mode and strength of interactions between birds and fruiting plants (Li et al. 2022). Despite these unique ecological conditions, little research has been conducted on the interaction network relationships between frugivorous birds and plants in karst habitats.

The Guangxi Chongzuo White‐headed Langur National Nature Reserve in subtropical southwest China represents a typical karst landscape. Human activities have transformed the scattered flatlands among the hills into large‐scale sugarcane plantations, further aggravating the natural habitat discontinuity between hills (Lu et al. 2021). Preliminary investigations have found that fruiting plants are scattered in fragmented patches of the protected area, often wrapped by alien farmlands, and birds feed on them during the fruit ripening period. Hypothesizing that unique frugivorous birds–plant interaction networks can form in fragmented karst habitats, we focused on the following research questions: (1) investigating the characteristics and seasonal variations of the frugivorous bird–plant interaction network structure in karst habitats; (2) identifying the most important frugivorous birds and plants in interaction networks; and (3) evaluating the possible impacts at the community level of both bird and plant species traits on their network functional roles. We predicted that the network structure would have seasonal differences, and the role of different species in the interaction network would be affected by both bird and plant species traits.

## 2 Materials and Methods

### 2.1 Study Area

This study was conducted in the Guangxi Chongzuo White‐headed Langur National Nature Reserve, Southwest Guangxi, China (107°17′–107°60′ E, 22°11′–22°37′ N). The reserve consists of four parts: Banli, Tuozhu, Bapen, and Dalin. Our research site is located in Banli (107°30′–107°31′, 22°15′–22°16′), with an area of 28.3 km^2^. This reserve features a typical karst forest with an altitude range of 400 to 600 m above sea level and exhibits marked seasonality in temperature, rainfall, and plant resource availability (Liu et al. 2022). The exceptionally high annual rainfall amounts to 4382.9 mm, and the mean daily temperature is 22.1°C. Extreme rainfall usually occurs with high temperatures, and the year can be divided into a dry season (October to March) and a rainy season (April to September) (Zhang et al. 2020). The vegetation profile is a limestone seasonal rain forest, with a small number of fleshy fruiting plants scattered at the foot of the mountain, such as *Ficus concinna*, *Sageretia thea* , and *Persicaria chinensis* .

### 2.2 Sampling of Frugivorous Bird–Plant Interactions

**FIGURE 1:**
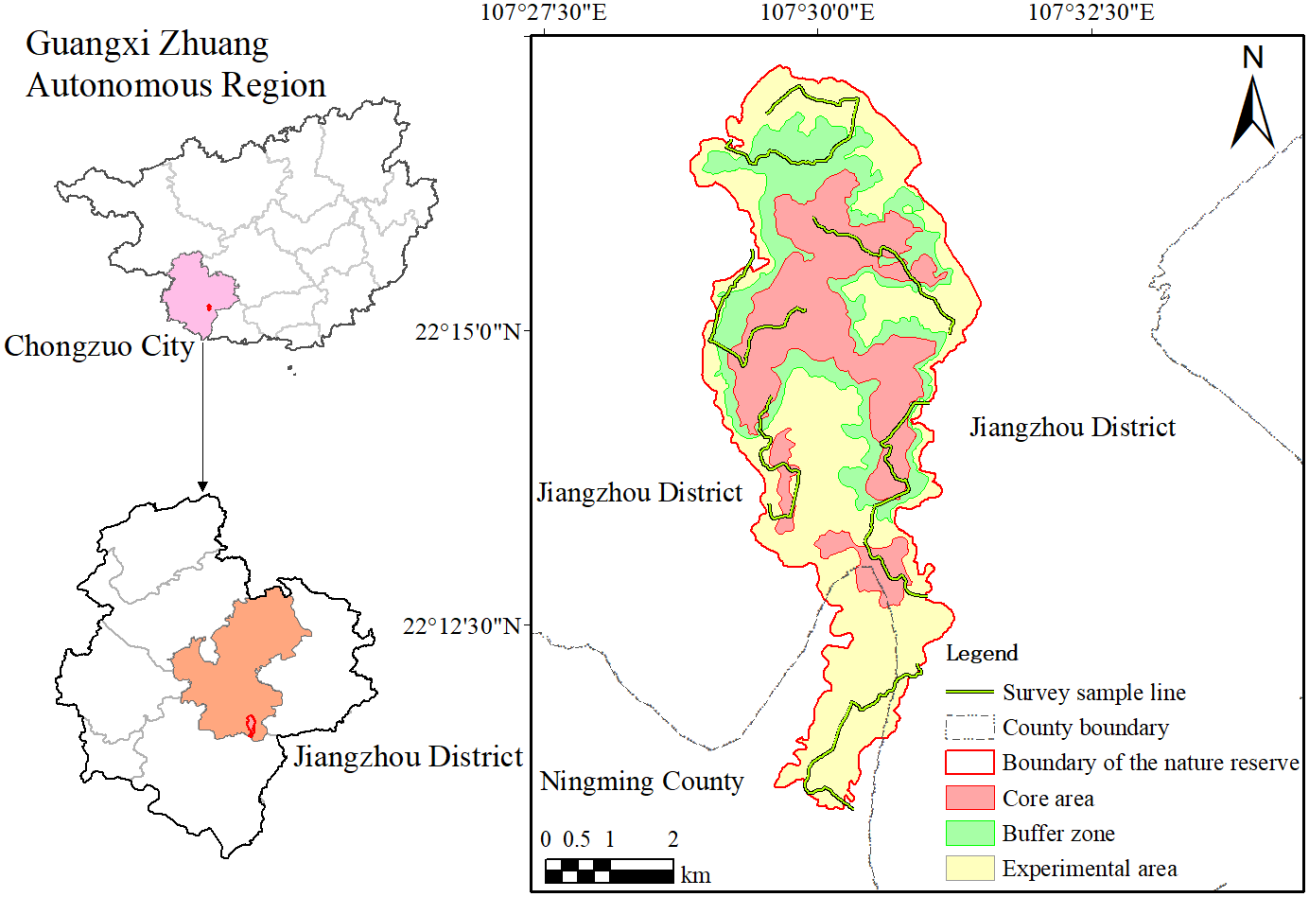
Distribution of the survey transects in Guangxi Chongzuo White‐headed Langur National Nature Reserve, southwest China.

Based on the terrain conditions and vegetation distribution characteristics in the survey area, we set up six transects of 3 to 5 km each to observe the foraging behavior of frugivorous birds from July 2023 to June 2024 (Figure 1). Foraging activities between frugivorous birds and target fruiting plants were observed by two experienced ornithologists with Safari l0 × 42 zoom binoculars in two foraging periods of 07:00 to 10:00 and 15:00 to 18:00 h. An interaction event was defined as a frugivorous bird landing on a fruiting plant's crown and eating at least one fruit before flying away (Breitbach et al. 2010). All six transects were surveyed once each week, accumulating 311.21 observation hours during the study period. Observation time per plant ranged from 3.37 to 71.50 h, with individual durations detailed in Supporting Information Table S1. Once birds were found foraging on plant fruits, the observation was conducted for at least 30 min to obtain sufficient foraging records. We recorded the bird and foraged plant species and the number of foraged fruits. The two experienced ornithologists conducted all the observations during the whole experimental period to control systematic errors. All the field surveys were carried out on calm days with no strong wind and no rain.

### 2.3 Fruit and Frugivorous Bird Traits

We collected morphological traits for all plant and bird species in the networks to identify the potential biological drivers underlying the network interaction role. For each plant species, we sampled fruit diameter (mm), fruit length (mm), fruit mass (g), fruit color, fruit abundance index (FAI), and fruit nutrition (sugar, fat, and protein). Vernier calipers and an electronic balance were employed to measure the length, diameter, and mass of 30 fruits per plant collected from three plants. Each fruiting individual was assigned a value of ripe FAI according to an ordinal scale: 0 = without fruits; 1 = 1 to 10 fruits; 2 = 11 to 100 fruits; 3 = 101 to 1000 fruits; 4 = 1001 to 10,000 fruits; and 5 > 10,000 fruits (Donoso et al. 2017; García et al. 2015). According to China's national standards, the nutrient contents of sugar (GB 5009.8‐2023), fat (GB 5009.6‐2016), and protein (GB 5009.3‐2016) were measured. For sugar content analysis, we used high‐performance liquid chromatography (1260‐IIPrime, Agilent Technologies Co. Ltd.) to quantify the types and proportions of sugar in the samples (Pizo et al. 2021). For fat content, we used the fat analyzer (SOX406, Haineng Future Technology Group Co. Ltd., Teodosio‐Faustino et al. 2021). For protein, we used the automatic Kjeldahl apparatus (K1160, Haineng Future Technology Group Co. Ltd.) to quantify the nitrogen content and multiply it by a fixed factor (6.25) to estimate the protein content (Maruyama et al. 2019). Five morphological bird traits, including bill width (mm), body length (mm), body mass (g), wing length (mm), and tail length (mm), were selected for their proven positively significant correlations with bird foraging types, seed dispersal distance, and visit frequency. These traits were studied following two key references: *A Field Guide to the Birds of China* (Mackinnon and Phillipps 2000) and *A Handbook of the Birds of China* (Zhao 2001).

### 2.4 Data Analysis

#### 2.4.1 Interaction Networks

In field observation, we found that birds pecked at a large fruit rather than swallowed the whole fruit, so the dispersal effectiveness of birds to fruiting plants cannot be assessed. To avoid the influence of different bird foraging patterns on the interaction network structure (Jordano 2016; Zhang et al. 2022), we constructed frugivorous bird–fruiting plant networks using the frequency of bird visits to plant species. The collected data were arranged as a matrix, with bird species as columns and plant species as rows, and each cell value represented the frequency of visits between each bird–plant pair.

The parameters of global topological patterns of local networks (such as richness metrics, connectance, nestedness, specialization, modularity, and robustness) can provide insight into the drivers of ecological processes and the robustness of ecological networks to changes and perturbations (Quitián et al. 2018). The relevant parameters are explained as follows: (1) Richness metrics, including the number of bird species, plant species, bird–plant links, and interaction frequency; (2) Connectance (*C*) varies from 0 (no interactions) to 1 (all species are connected to each other). It is the proportion of recorded connections with respect to the total number of potential connections. It is based on the formula *C* = *I*/(*A* × *B*), where *I* = the number of observed interactions, *A* = the number of bird species, and *B* = the number of plant species with fruits detected in the study (Fernández and Fontúrbel 2021); (3) Weighted nestedness (a metric based on the overlap and decreasing fill, *w*NODF), denotes the ratio between the mean number of interactions per species and the total number of species in the network (Zhang et al. 2022). When *w*NODF is close to 0, there is no evidence of aggregation in the matrix. When it approaches 100, the interactions are increasingly nested (Gonzalez and Loiselle 2016); (4) Specialization (*H*_2_ *´*) measures the niche complementarity between species and integrates specializations across the entire community. It ranges from 0 to 1. When *H*_2_´ is close to 1, the interactions reflect a high degree of specialization (Blüthgen et al. 2006; Wang et al. 2023); (5) Modularity (*Q*) varies from 0 (no modularity) to 1 (network is organized into modules). It identifies cohesive subgroups of species within the network that interact strongly among themselves, usually associated with trait matching, functional groups, and/or habitat and diet requirements in ecological studies (Dormann and Strauss 2014; Pinto et al. 2021); (6) Robustness (*R*) represents the stability of the network when a given set of species is eliminated from the recorded network (Bascompte and Jordano 2007). We used two different methods based on species degree (cf. Section 2.4.2), eliminating species one by one from the highest to the lowest degree and vice versa. Simulated extinction scenarios (i.e., removal from the network) for both plant and bird species based on these two methods were compared with a null model scenario based on the random deletion of species (Memmott et al. 2004). *R* ranges from 0 to 1, with a value approaching 1 indicating greater network stability after species loss or removal (Hernández‐Dávila et al. 2022).

To test whether the observed network parameters differ from those generated by null models, we generated 1000 randomized matrices using the r2dtable algorithm in the null model based on the original weighted matrix. This method randomizes the interactions among species while maintaining network size and marginal totals consistent with the original matrix (Pigot et al. 2016; Vaughan et al. 2018). Significance (*p*) was estimated as the number of times the null model resulted in a network with a score equal to or higher than the score measured in the original matrix, divided by the total number of randomizations (Santos and Ribeiro 2023). All analyses were performed by the “network‐level” function in the “bipartite” package version 4.3.3 (R Development Core Team 2024), and the significant difference was set at *p* < 0.05.

Due to differences in richness data and interaction heterogeneity, the network parameter values (connectance, weighted nestedness, specialization, and modularity) were standardized to facilitate their comparison. To this effect, their *Z*‐scores were computed: *Z* = (Obs‐Exp_null(1…*n*)_)/Sd_null_, where Obs is the observed value, Exp_null(1…*n*)_, and Sd_null_ are the mean value and standard deviation of 1000 randomizations derived from the null model, respectively (Ulrich and Gotelli 2007).

We used individual‐based extrapolation/interpolation methods to determine the sample coverage for bird and plant species, as well as bird–plant interactions (Li et al. 2022). We treated ‘abundance’ as the interaction frequencies recorded for each pairwise link and performed this analysis using the *i*NEXT function in the R package ‘*i*NEXT’ v2.0.20 (Hsieh et al. 2016).

#### 2.4.2 Relationships Between Species' Traits and Their Functional Roles

To quantify the contribution of both bird and plant species to the network topological pattern, we calculated three species‐level metrics that take into account the most important organization in bird–plant interaction networks: (1) Species degree for both plants and birds quantifies the number of links of each species (Angulo‐Ortiz et al. 2024; Zhu et al. 2024); (2) species strength represents the sum of the dependencies of each species' relevance across all partners (Bascompte and Jordano 2007); (3) specialization for both plants and birds quantifies species selectivity in relation to resource availability determined by the marginal totals of the observed interaction matrix (Costa et al. 2020; Spotswood et al. 2012).

Given the significant differences in scale between variables, we applied a log10 transformation to all data to improve analysis linearity. Subsequently, one‐sample Kolmogorov–Smirnov testing on the transformed data confirmed normality for all variables. Generalized linear models (GLMs; “lme4” package) with Gaussian error distribution were applied to estimate the effect of species traits on their network role. Model averaging (dredge and model.avg. functions in the “MuMIn” package) then evaluated and synthesized the results, with model selection based on the Akaike Information Criterion (AIC). We set the transformed network parameters (species degree, species strength, and specialization) as the response variable, with transformed bird and plant traits as explanatory variables. Bird traits included bill width, body length, body mass, wing length, and tail length. Plant traits included fruit diameter, fruit length, fruit mass, fruit color, FAI, and fruit nutrition (sugar, fat, and protein). The analyses were conducted using R version 4.3.3 (R Development Core Team 2024). All tests were two‐tailed, with significance levels of 0.05.

## 3 Results

### 3.1 The Bird–Plant Interaction Network

**FIGURE 2:**
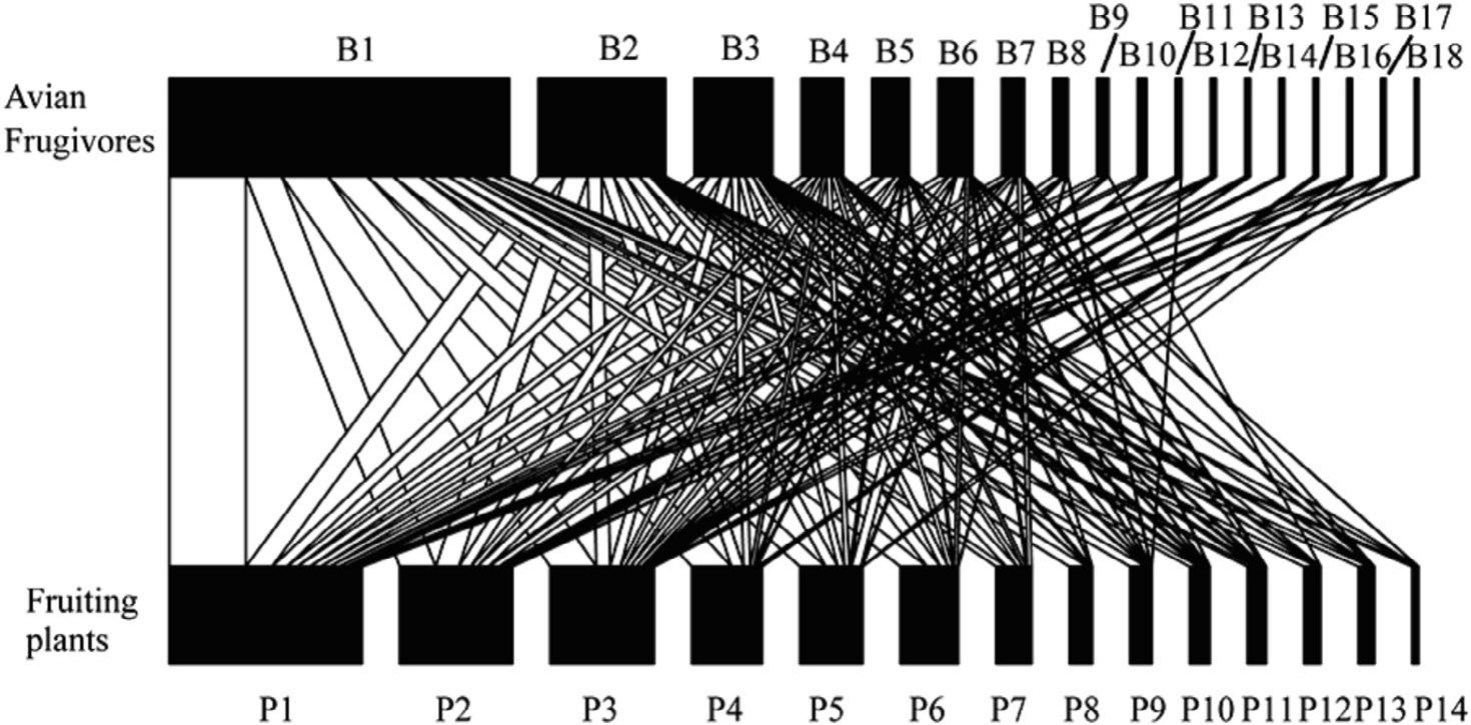
Diagram of the plant–frugivorous bird interaction network in the karst habitat. B1 to B18 represents 18 species of frugivorous birds, and P1 to P14 represents 14 fruiting plant species. See Table 2 for corresponding Latin names.

A total of 4446 interactions were observed (Figure 2). The interaction web contained 14 plant species (10 families) and 18 frugivorous bird species (10 families). Each plant species had, on average, 7.86 ± 0.94 interactions (mean ± SD, ranging from 4 to 14) with birds. Each bird species had, on average, 6.11 ± 1.15 interactions (ranging from 1 to 14) with plants. The plant species with the highest degree were *F. concinna*, *Cinnamomum camphora* , and *F. altissima* , each interacting with 14, 11, and 16 bird species, respectively. Frugivorous birds with the highest degree were *P. jocosus* , *P. aurigaster* , and *P. sinensis* , each interacting with 14 plant species.

**FIGURE 3:**
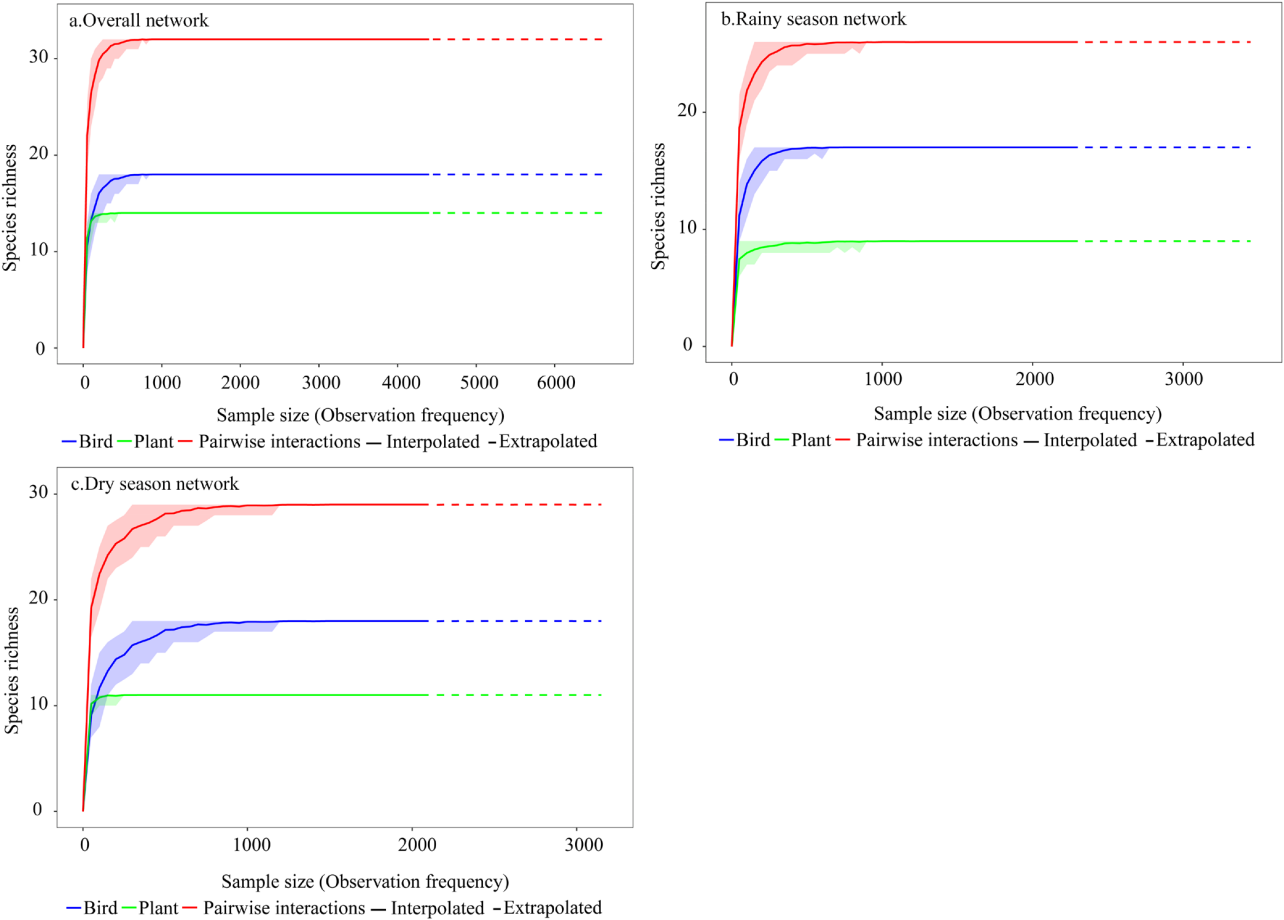
Sampling coverage of the plant–frugivorous bird network in the karst habitat. Observed (interpolated, solid line) and simulated (extrapolated, dashed line) data for paired interactions (blue), plants (green), and birds (red) are well‐represented by the sampling coverage across (a) overall network, (b) rainy season network, and (c) dry season network.

Sample coverage of bird–plant interactions reached 99.74% in the overall network (birds: 99.48%; plants: 99.17%), 99.83% in the rainy season (birds: 99.35%; plants: 99.66%), and 99.82% in the dry season (birds: 99.76%; plants: 98.26%), indicating sufficient sampling of interactions without seasonal bias in completeness (Figure 3a–c).

**FIGURE 4:**
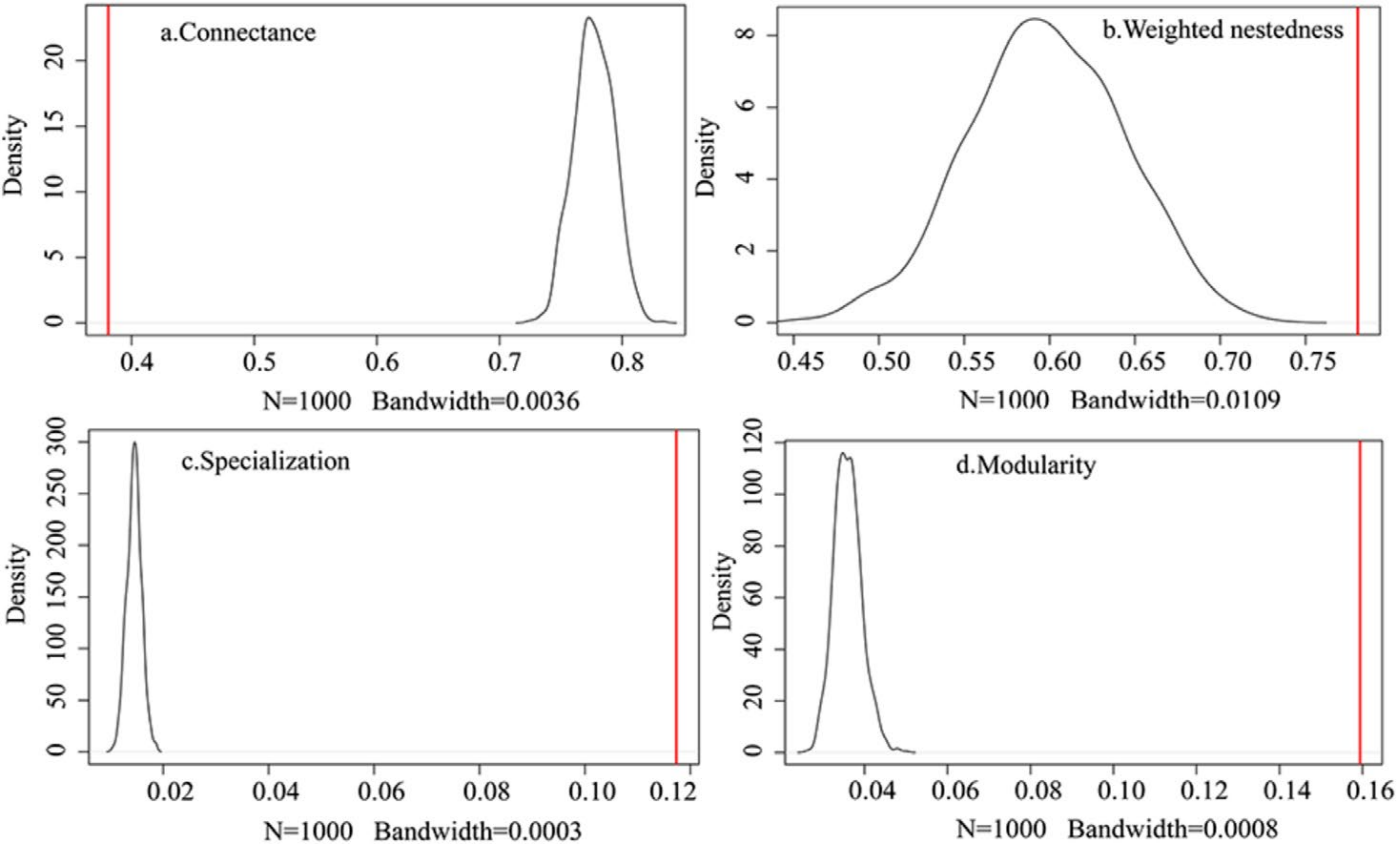
Comparison between the observed network and the networks generated by the null model. (a): Connectance; (b): Weighted nestedness; (c): Specialization; (d): Modularity. The red line represents the parameter values of the actual observed network matrix, and the black line represents the parameter values of the network randomly generated by the null model.

At the network level, the observed network structure showed a lower connectance (*C*_*z*‐score_ = −24.86, *p* < 0.01), higher specialization (*H*_2_′_*z*‐score_ = 75.39, *p* < 0.01), and higher modularity (*Q*_*z*‐score_ = 35.85, *p* < 0.01) than the random networks produced by the null model (*N* = 1000; Table 1 and Figure 4). The weighted nestedness value was higher than, but not significantly different from, the values in randomized networks (*w*NODF_*z*‐score_ = −1.34, *p* = 0.179). Considering the weather effect, the rainy season contributed 52.25% of the annual network connections and the dry season 47.75% (Table 1). In the overall network, 17 bird species and 6 plant species were present in both seasons. The connectance, weighted nestedness, specialization, and modularity were higher in rainy than in dry seasons (Table 1). Compared with the random networks produced by the null model, weighted nestedness demonstrated no significant difference in rainy seasons (*p* = 0.78).

**TABLE 1:** Metrics characterizing the interaction networks between frugivorous birds and plants in karst habitats.

Parameter	Overall	Rainy season	Dry season
Bird richness	18	17	18
Plant richness	14	9	11
Number of links	110	77	85
Interaction frequency	4446	2323	2123
Connectance(*C*_*z‐score*_)	−24.86**	−15.65**	−17.54**
Weighted nestedness (*w*NODF_*z*‐score_)	−1.34	−0.280	−2.59**
Specialization (*H*_2_´_*z*‐score_)	75.39**	47.70**	40.31**
Modularity (*M*_*z*‐score_)	35.85**	25.01**	23.04**

** *p* < 0.01.

**FIGURE 5:**
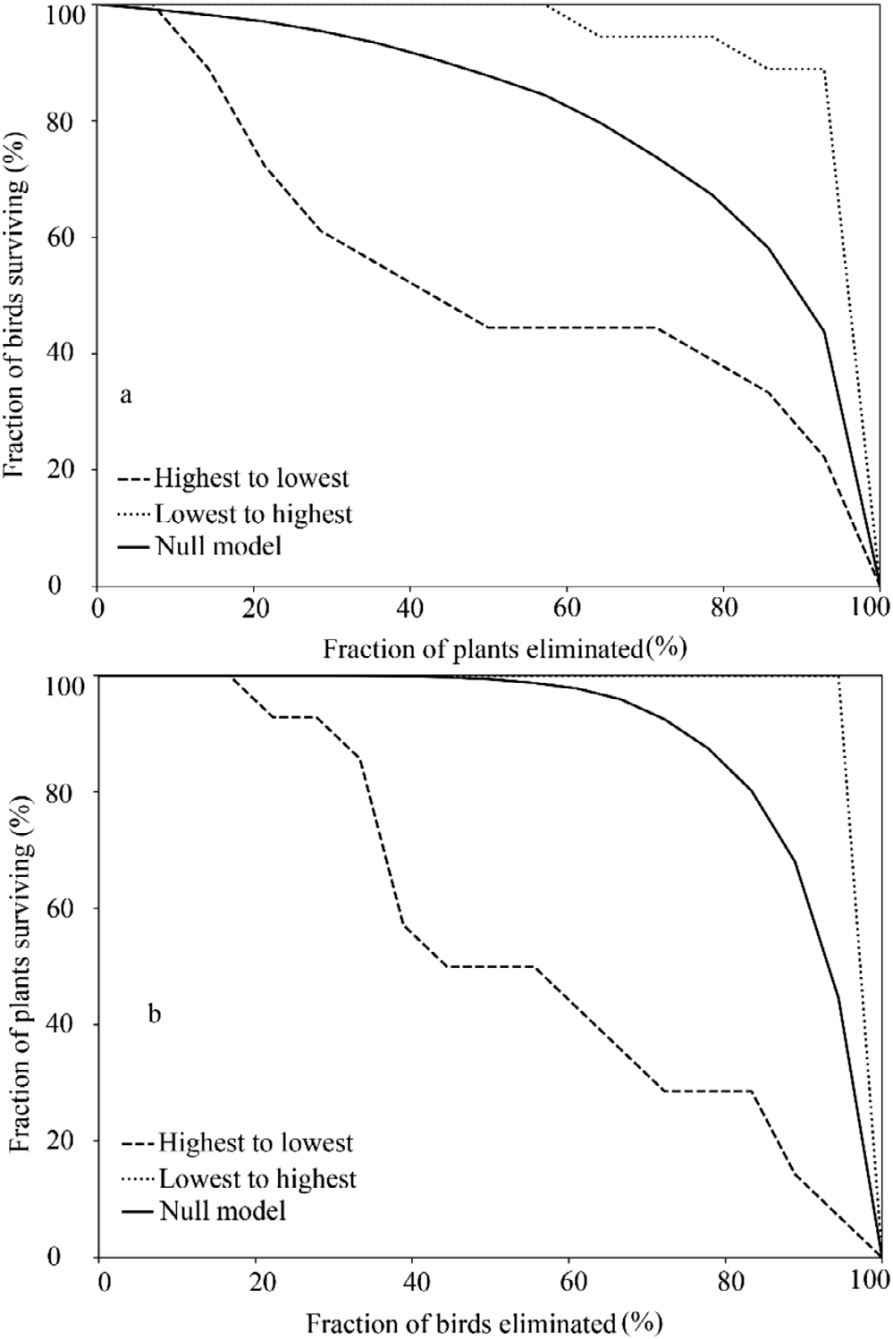
Simulation scenarios of extinction or elimination of bird or plant species in the interaction network in karst habitats using two methods: from highest to lowest species degree (dashed line) and from lowest to highest species degree (dotted line). The null model is shown as a solid line. The *x*‐axis represents eliminated bird or plant species (%), and the *y*‐axis represents surviving bird or plant species (%).

Network robustness under the null random model was *R* = 0.845 for birds and *R* = 0.689 for plants. In the scenario of species eliminated from highest to lowest degree, robustness was *R* = 0.459 for birds and *R* = 0.404 for plants. Eliminating species from the lowest to highest degree, robustness was *R* = 0.936 for birds and *R* = 0.872 for plants. Extinction scenario simulations showed that after eliminating 40% of the most connected species, only 50% to 60% of the bird or plant species would remain in the network. In contrast, after eliminating 40% of the less connected species, almost all interacting bird and plant species would remain in the network. Finally, the null model showed that when 40% of the bird or plant species were eliminated randomly from the network, approximately 90% of the birds and almost all plant species would remain in the network (Figure 5).

### 3.2 Relationship Between Species' Traits and Functional Roles

The bird species ( *P. jocosus* , *P. aurigaster* , and *P. sinensis* ) and plant species ( *F. concinna* , *C. officinarum* , and *F. altissima* ) simultaneously have the highest species degree and species strength (Table 2), indicating that these species had the most central position relative to the other species in the network; this is because they participated in the most frequent interaction of all those recorded.

**TABLE 2:** Species‐level metrics for the bird–plant frugivory interaction network in karst habitats.

Code	Species	Species degree	Species strength	Specialization
*Bird species*				
B1	*Pycnonotus jocosus*	14	7.527	0.048
B2	*Pycnonotus aurigaster*	14	2.241	0.037
B3	*Pycnonotus sinensis*	14	1.557	0.013
B4	*Pycnonotus xanthorrhous*	11	0.668	0.040
B5	*Phylloscopus inornatus*	10	0.432	0.069
B6	*Zosterops japonicus*	11	0.547	0.124
B7	*Orthotomus sutorius*	8	0.374	0.160
B8	*Spilopelia chinensis*	4	0.173	0.292
B9	*Copsychus saularis*	3	0.081	0.228
B10	*Lonchura punctulata*	2	0.056	0.221
B11	*Passer montanus*	3	0.052	0.178
B12	*Turdus merula*	3	0.043	0.143
B13	*Spizixos semitorques*	2	0.049	0.250
B14	*Spodiopsar cineraceus*	1	0.045	0.378
B15	*Acridotheres cristatellus*	1	0.070	0.470
B16	*Spodiopsar sericeus*	4	0.037	0.144
B17	*Dicaeum cruentatum*	3	0.026	0.214
B18	*Lonchura striata*	2	0.022	0.206
*Plant species*				
P1	*Ficus concinna*	14	6.802	0.093
P2	*Camphora officinarum*	11	4.149	0.167
P3	*Ficus altissima*	16	2.653	0.024
P4	*Melia azedarach*	7	1.474	0.108
P5	*Flueggea virosa*	9	0.838	0.068
P6	*Bischofia javanica*	7	0.586	0.053
P7	*Phyllanthus reticulatus*	6	0.494	0.106
P8	*Ficus tinctoria*	5	0.119	0.108
P9	*Broussonetia papyrifera*	8	0.231	0.077
P10	*Causonis japonica*	6	0.189	0.050
P11	*Maclura tricuspidata*	6	0.224	0.070
P12	*Syzygium cumini*	5	0.100	0.106
P13	*Persicaria chinensis*	6	0.105	0.059
P14	*Sageretia thea*	4	0.038	0.068

Body mass showed a significant negative correlation with species degree (*β* = −3.130, *p* = 0.01) and species strength (*β* = −5.111, *p* = 0.032), but a positive correlation with specialization (*β* = 3.829, *p* = 0.006; Table 3). Body length showed a positive correlation with species degree (*β* = 7.728, *p* = 0.01) and a negative correlation with specialization (*β* = −10.332, *p* = 0.045; Table 3). Fruit abundance index (FAI) was the only plant trait correlated positively with species degree (*β* = 0.986, *p* = 0.004) and species strength (*β* = 4.391, *p* < 0.001; Table 4), indicating that plants with a higher fruit abundance had greater bird species degree and species strength.

**TABLE 3:** Results of generalized linear models (GLM) evaluating the effects of bird traits on their network role.

Variable	Estimate	Standard error	*Z*‐value	*p*
*Species degree*				
Intercept	−10.608	5.003	2.034	0.042
Body length	7.728	2.807	2.576	0.010*
Body mass	−3.130	1.019	2.898	0.004**
Bill width	−0.623	0.986	0.583	0.560
Wing length	1.415	2.450	0.538	0.591
Tail length	1.023	2.079	0.473	0.636
*Species strength*				
Intercept	−18.219	12.243	1.447	0.148
Body length	13.954	7.167	1.850	0.064
Body mass	−5.111	2.249	2.151	0.032*
Bill width	−2.106	2.152	0.907	0.365
Wing length	−1.748	5.178	0.317	0.751
Tail length	1.677	4.322	0.374	0.709
*Specialization*				
Intercept	14.155	6.832	1.991	0.046
Body length	−10.332	4.924	2.007	0.045*
Body mass	3.829	1.329	2.725	0.006*
Bill width	0.862	1.296	0.620	0.535
Wing length	−1.914	2.743	0.648	0.517
Tail length	2.150	2.604	0.788	0.431

* 0.01 < *p* < 0.05.

** *p* < 0.01.

**TABLE 4:** Results of generalized linear models (GLM) evaluating the effects of plant traits on their network role.

Variable	Estimate	Standard error	*Z*‐value	*p*
*Species degree*				
Intercept	0.381	0.210	1.688	0.091
Fruit length	−0.041	0.185	0.199	0.842
Fruit diameter	−0.057	0.151	0.342	0.732
Fruit mass	0.037	0.076	0.440	0.660
Fruit color	0.042	0.185	0.210	0.834
FAI	0.986	0.305	2.881	0.004**
Protein	0.012	0.278	0.039	0.969
Fat	0.200	0.165	1.078	0.281
Sugar	−0.016	0.047	0.299	0.765
*Species strength*				
Intercept	−2.696	0.587	4.189	< 0.001
Fruit length	−0.385	0.442	0.773	0.440
Fruit diameter	−0.189	0.421	0.398	0.691
Fruit mass	−0.167	0.185	0.795	0.427
Fruit color	0.285	0.547	0.474	0.635
FAI	4.391	0.963	4.103	< 0.001**
Protein	1.018	0.753	1.210	0.226
Fat	0.944	0.448	1.874	0.061
Sugar	−0.028	0.145	0.171	0.864
*Specialization*				
Intercept	−1.156	0.192	5.492	< 0.001
Fruit length	−0.033	0.304	0.098	0.922
Fruit diameter	−0.071	0.218	0.290	0.771
Fruit mass	−0.099	0.119	0.749	0.454
Fruit color	−0.052	0.241	0.193	0.847
FAI	0.058	0.565	0.092	0.927
Protein	0.498	0.383	1.159	0.247
Fat	0.225	0.279	0.722	0.471
Sugar	0.009	0.083	0.092	0.926

** *p* < 0.01.

## 4 Discussion

This is the first study to analyze interaction networks between frugivorous birds and fruiting plants in fragmented karst habitats. Compared to the interaction networks of other fragmented habitats (Montoya‐Arango et al. 2019; Li et al. 2022), the karst habitats exhibit simpler species composition. This difference may be attributed to notable shifts in landscape patterns in karst habitats. The natural habitat fragmentation has been accentuated by large‐scale clearing of natural vegetation for sugarcane cultivation, reducing landscape connectivity, increasing habitat isolation, and diminishing fruit diversity (Liu et al. 2022). Passerine birds are the main component of the observed interaction network. As generalist frugivorous with a smaller size, they supplement their insect and invertebrate diet with fruits and seeds and establish large populations (Cramer et al. 2007). Furthermore, they are endowed with ecological versatility, including different body traits, broad distribution, and high mobility that collectively enable them to occupy central positions within the network (Wang et al. 2023).

The observed network connectance is simpler than the random network produced by the null model. This result could be attributed to the random network constructed by the null model that assumes an equal probability of connections between all species (Costa et al. 2020). The intrinsic mathematical behavior of the null model generates more connected matrices than most observed networks (Wang et al. 2023). Weighted nestedness did not differ from the random network, suggesting that the few specialist interactions were not nested within the more generalist network structure (Chiew et al. 2022). The degree of specialization and modularity of the observed interaction network was significantly higher than that of the null model. The high specialization level may be related to birds' behavioral responses to environmental degradation and reduced fruit resource availability (González‐Castro et al. 2015). Previous studies have shown that decreasing resource availability can increase interspecific competition among coexisting species, enhance specialization, and reduce niche overlap (Tinoco et al. 2017). Moreover, severe habitat fragmentation in karst landscapes reduces patch connectivity, decreasing fruit resources and aggravating their uneven spatial distribution. Furthermore, highly specialized species are vulnerable to disappearance from the networks, particularly in karst habitats that are highly disturbed and severely fragmented. The extinction of one species may usher in fatal collateral consequences for its interaction partners (Sebastián‐González et al. 2015). Higher modularity in interaction networks is more common when networks include different niches, syndromes, or species in distantly related clades (Pinheiro et al. 2022). Other studies have also shown that interaction networks between frugivorous birds and fruiting plants in fragmented habitats exhibit high modularity, enhancing their resilience to environmental changes (Li et al. 2022).

We found seasonal variations in the structure of the interaction network, which is consistent with our first prediction. The network's connectance was higher in the rainy season than in the dry season. This phenomenon could be explained by the rainy season's higher abundance and availability of fruit resources than the dry season (Chen et al. 2023). Additionally, suitable environmental conditions, strong species activities, and high reproductive demands in the rainy season collectively bring higher connectance (Santos and Ribeiro 2023; Zhu et al. 2024). Considering that weighted nestedness and modularity are often associated with network stability and reduction of interspecific competition, higher values during the rainy season may foster the persistence of species that are less abundant or more selective in their resource use (Fortuna et al. 2010; Donatti et al. 2011). Higher specialization in the rainy season indicates more niche differentiation and efficient resource utilization (Sebastián‐González et al. 2015). However, it also makes species more vulnerable to environmental changes or the loss of interaction partners (Silva et al. 2016). The seasonal changes in these indicators reflect the dynamic response of the bird‐fruit interaction network to karst environmental conditions and the complexity and adaptability of species interactions.

In various scenarios of simulated selective extinction, the maximum decrease in network stability typically occurs after removing the most closely related species, with plants the most affected. Plants can provide habitat, food, and other resources for various bird species in ecosystems, and this clinching fundamental role probably determines the above response (Hernández‐Dávila et al. 2022). However, in karst habitats, the high‐intensity human disturbances have led to widespread clearance of natural vegetation for crops (e.g., sugarcane, maize, and cassava), resulting in significant plant species loss. Such habitat transformation may trigger collateral effects and cascading ecological impacts that ultimately compromise ecosystem structure and function (Li et al. 2022).

The bird species that contributed most to the interaction network were *P. jocosus* , *P. aurigaster* , and *P. sinensis* . These three resident bird species are generally dominant in karst habitats, demonstrating a wider range of ecological tolerance than other species to habitat fragmentation and foraging on most fruiting plants. Therefore, they play a key role as seed dispersers and maintain the stability of the network structure in karst habitats (Sebastián‐González 2017). Among plant species, *Ficus altissima* , *F. concinna* , and *Camphora officinarum* registered the highest species strength. These species have sweet fruits and maintain high fruit‐setting rates, rendering them attractive to frugivorous birds. We recommend prioritizing these species for large‐scale planting in karst habitats to enhance network complexity and biodiversity.

Significant associations were observed between species traits and network roles, consistent with previous study results (Saavedra et al. 2014; Pigot et al. 2016) and our second prediction. Our study found that the bird's body mass had a significant negative correlation with species degree and species strength and a positive correlation with specialization. The differences in bird species traits and functional roles incur the interspecific divergence in fruit resource utilization (Dehling et al. 2016). Body mass determines energy demand and preferred fruit crop size (Albrecht et al. 2018). According to the optimal foraging theory, large‐bodied birds preferentially forage on plants with substantial crop mass‐concentrated resource patches that provide high‐energy rewards while minimizing foraging effort (Wotton and Kelly 2012; Dehling et al. 2016). The selective foraging of large‐bodied birds on energy‐dense resources reduces their species degree and species strength within plant‐frugivorous networks and leads to significant specialization (González‐Castro et al. 2015). Moreover, the disappearance of large birds from small habitat patches is one of the most pervasive impacts of habitat fragmentation. This is because birds with larger body mass have larger area requirements, lower population density, and reproductive output, as well as being the target of increased human‐related activities like hunting in fragmented environments (García et al. 2015).

The bird's body length can shape network species degree and specialization, which can, in turn, influence the structure and functioning of ecological networks (Wang et al. 2023). Body length was positively correlated with species degree and negatively associated with specialization. Body length can affect the ability to acquire resources, niche breadth, mobility, and social behavior (Schleuning et al. 2011). For instance, birds with larger body lengths have stronger flight abilities and higher intra‐vegetation mobility (Wang et al. 2023). Such qualities facilitate scouting for diverse fruit resources and forming more interaction relationships with fruiting plants across different trophic levels and spatial scales (Acevedo‐Quintero et al. 2020). Furthermore, birds with larger body lengths may develop more complex social behaviors or foraging strategies, allowing specific foraging of fruit resources with certain characteristics, such as specialized feeding on large fruits, making the network structure more specialized (Quintero et al. 2020).

FAI is significantly positively correlated with species degree and species strength, crucial in maintaining network stability and biodiversity (Cousens et al. 2010). Fruit supply shapes the population size of birds, inter‐specific competition, and interaction within the network (Villegas et al. 2024). Many tropical and subtropical plants have a long and asynchronous fruiting period, producing fruits at different stages and times of the year, with different traits, nutrition, and energy to meet the varied food needs of frugivorous birds in various ecological niches (Wang et al. 2023). Such an assortment of plant phenology and physiology can increase the variety and quantity of frugivorous birds through several mechanisms. They include reducing competition for food resources between frugivorous birds, increasing network connectivity and complexity, and allowing more species to coexist (Quintero et al. 2020). In the study area, *F. altissima* , *F. concinna*, and *C. camphora* exhibited the highest fruiting frequencies, providing abundant food resources at different times of the year. Their generous and time‐intensive food provision for diverse bird species is disproportionately important in maintaining network stability. On the contrary, the decrease in fruit availability can incur temporal and spatial changes in composition and behavior in frugivorous birds and simplify the network structure (Ramos‐Robles et al. 2016). Such alterations can dampen the regeneration of plant populations and ecosystem service functions that rely on frugivorous birds as dispersal media.

## 5 Conclusions

Our results demonstrate that complex interaction networks can form between frugivorous birds and fruiting plants in spatially fragmented and degraded karst habitats. These findings provide fundamental data and background for future quantified investigations of how the patchy, disjointed karst habitat patches affect bird–plant interaction networks. The interaction network in this study was constructed from one year of bird foraging data. Since feeding does not necessarily correlate with effective seed dispersal or other ecological functions, long‐term monitoring of interactions and assessment of bird foraging behavior, bird abundance, and plant phenology will deepen understanding of bird–plant interactions in karst habitat. The continual knowledge accumulation may improve the capacity to conserve and restore the karst landscapes.

## Author Contributions

**Guohai Wang:** data curation (equal), funding acquisition (equal), investigation (equal), writing – original draft (equal). **Yanru Wang:** data curation (equal). **Can Zhou:** investigation (equal). **Huangmin Zhang:** investigation (equal). **Lijuan Wei:** investigation (equal). **Dengpan Nong:** investigation (equal). **Chi Yung Jim:** supervision (equal), writing – review and editing (equal). **Qihai Zhou:** funding acquisition (equal), supervision (equal), writing – review and editing (equal).

## Conflicts of Interest

The authors declare no conflicts of interest.

## Supporting information


**Table S1:** Observation time per plant species: Overall, rainy season, and dry season.

## Data Availability

The complete dataset for this research is available in the data repository Dryad (https://doi.org/10.5061/dryad.crjdfn3fw).

## References

[ece372087-bib-0001] Acevedo‐Quintero, J. F. , J. G. Zamora‐Abrego , and D. García . 2020. “From Structure to Function in Mutualistic Interaction Networks: Topologically Important Frugivores Have Greater Potential as Seed Dispersers.” Journal of Animal Ecology 89, no. 9: 2181–2191. 10.1111/1365-2656.13273. 32495479

[ece372087-bib-0002] Albrecht, J. , A. Classen , M. G. R. Vollstädt , et al. 2018. “Plant and Animal Functional Diversity Drive Mutualistic Network Assembly Across an Elevational Gradient.” Nature Communications 9, no. 1: 3177. 10.1038/s41467-018-05610-w. PMC6085337 30093613

[ece372087-bib-0003] Angulo‐Ortiz, D. , J. Becoche‐Mosquera , and L. G. Gómez‐Bernal . 2024. “Structure of Plant‐Frugivorous Bird Interaction Networks in Two High Andean Forests of Southwestern Colombia.” Global Ecology and Conservation 55: e03254. 10.1016/j.gecco.2024.e03254.

[ece372087-bib-0004] Banks‐Leite, C. , R. M. Ewers , and J. P. Metzger . 2012. “Unraveling the Drivers of Community Dissimilarity and Species Extinction in Fragmented Landscapes.” Ecology 93, no. 12: 2560–2569. 10.1890/11-2054.1. 23431587

[ece372087-bib-0005] Bascompte, J. , and P. Jordano . 2007. “Plant‐Animal Mutualistic Networks: The Architecture of Biodiversity.” Annual Review of Ecology, Evolution, and Systematics 38: 567–593. 10.1146/annurev.ecolysys.38.092106.095818.

[ece372087-bib-0006] Blendinger, P. G. , N. P. Giannini , I. C. Zampini , et al. 2015. “Nutrients in Fruits as Determinants of Resource Tracking by Birds.” Ibis 157: 480–495. 10.1111/ibi.12274.

[ece372087-bib-0007] Blüthgen, N. , F. Menzel , and N. Blüthgen . 2006. “Measuring Specialization in Species Interaction Networks.” BMC Ecology 6: 1–12. 10.1186/1472-6785-6-9. 16907983 PMC1570337

[ece372087-bib-0008] Breitbach, N. , I. Laube , I. Steffan‐Dewenter , and K. Böhning‐Gaese . 2010. “Bird Diversity and Seed Dispersal Along a Human Land‐Use Gradient: High Seed Removal in Structurally Simple Farmland.” Oecologia 162: 965–976. 10.1007/s00442-009-1547-y. 20049479

[ece372087-bib-0009] Cazetta, E. , and L. Fahrig . 2022. “The Effects of Human‐Altered Habitat Spatial Pattern on Frugivory and Seed Dispersal: A Global Meta‐Analysis.” Oikos 2: e08288. 10.1111/oik.08288.

[ece372087-bib-0010] Chen, Y. , Y. Lai , J. Zheng , et al. 2023. “Seasonal Variations in the Gut Microbiota of White‐Headed Black Langur (*Trachypithecus leucocephalus*) in a Limestone Forest in Southwest Guangxi, China.” Frontiers in Ecology and Evolution 11: 1126243. 10.3389/fevo.2023.1126243.

[ece372087-bib-0011] Chiew, L. Y. , T. D. Hackett , J. F. Brodie , et al. 2022. “Tropical Forest Dung Beetle‐Mammal Dung Interaction Networks Remain Similar Across an Environmental Disturbance Gradient.” Journal of Animal Ecology 91: 604–617. 10.1111/1365-2656.13655. 34954816

[ece372087-bib-0012] Costa, J. M. , J. A. Ramos , S. Timóteo , L. P. da Silva , R. S. Ceia , and R. H. Heleno . 2020. “Species Temporal Persistence Promotes the Stability of Fruit‐Frugivore Interactions Across a 5‐Year Multilayer Network.” Journal of Ecology 108, no. 5: 1888–1898. 10.1111/1365-2745.13391.

[ece372087-bib-0013] Cousens, R. D. , J. Hill , K. French , and I. D. Bishop . 2010. “Towards Better Prediction of Seed Dispersal by Animals.” Functional Ecology 24, no. 6: 1163–1170. 10.1111/j.1365-2435.2010.01747.x.

[ece372087-bib-0014] Cramer, J. M. , R. C. G. Mesquita , and G. B. Williamson . 2007. “Forest Fragmentation Differentially Affects Seed Dispersal of Large and Small‐Seeded Tropical Trees.” Biological Conservation 137, no. 3: 415–423. 10.1016/j.biocon.2007.02.019.

[ece372087-bib-0015] Dehling, D. M. , P. Jordano , H. M. Schaefer , K. Böhning‐Gaese , and M. Schleuning . 2016. “Morphology Predicts Species' Functional Roles and Their Degree of Specialization in Plant‐Frugivore Interactions.” Proceedings of the Royal Society B: Biological Sciences 283: 20152444. 10.1098/rspb.2015.2444. PMC4795026 26817779

[ece372087-bib-0016] Dehling, D. M. , T. Töpfer , H. M. Schaefer , P. Jordano , K. Böhning‐Gaese , and M. Schleuning . 2014. “Functional Relationships Beyond Species Richness Patterns: Trait Matching in Plant‐Bird Mutualisms Across Scales.” Global Ecology and Biogeography 23, no. 10: 1085–1093. 10.1111/geb.12193.

[ece372087-bib-0017] Donatti, C. I. , P. R. Guimarães , M. Galetti , M. A. Pizo , F. M. D. Marquitti , and R. Dirzo . 2011. “Analysis of a Hyper‐Diverse Seed Dispersal Network: Modularity and Underlying Mechanisms.” Ecology Letters 14: 773–781. 10.1111/j.146-0248.2011.01639.x. 21699640

[ece372087-bib-0018] Donoso, I. , D. García , D. Martínez , J. M. Tylianakis , and D. B. Stouffer . 2017. “Complementary Effects of Species Abundances and Ecological Neighborhood on the Occurrence of Fruit‐Frugivore Interactions.” Frontiers in Ecology and Evolution 5: 133. 10.3389/fevo.2017.00133.

[ece372087-bib-0019] Dormann, C. F. , and R. Strauss . 2014. “A Method for Detecting Modules in Quantitative Bipartite Networks.” Methods in Ecology and Evolution 5, no. 1: 90–98. 10.1111/2041-210X.12139.

[ece372087-bib-0020] Fernández, V. P. , and F. E. Fontúrbel . 2021. “Temporal Variation of Daily Activity on Pollinator and Frugivorous Birds Simultaneously Interacting With a Specialized Mistletoe.” Community Ecology 22: 217–223. 10.1007/s42974-021-00050-x.

[ece372087-bib-0021] Fortuna, M. A. , D. B. Stouffer , J. M. Olesen , et al. 2010. “Nestedness Versus Modularity in Ecological Networks: Two Sides of the Same Coin?” Journal of Animal Ecology 79: 811–817. 10.1111/j.1365-2656.2010.01688.x. 20374411

[ece372087-bib-0022] García, D. , D. Martínez , D. B. Stouffer , and J. M. Tylianakis . 2015. “Exotic Birds Increase Generalization and Compensate for Native Bird Decline in Plant‐Frugivore Assemblages.” Journal of Animal Ecology 83: 1441–1450. 10.1111/1365-2656.12237. 24749667

[ece372087-bib-0023] Gonzalez, O. , and B. A. Loiselle . 2016. “Species Interactions in an Andean Bird‐Flowering Plant Network: Phenology Is More Important Than Abundance or Morphology.” PeerJ 4: e2789. 10.7717/peerj.2789. 27994982 PMC5157195

[ece372087-bib-0024] González‐Castro, A. , Y. Suann , M. Nogales , and T. A. Carlo . 2015. “Relative Importance of Phenotypic Trait Matching and Species' Abundances in Determining Plant‐Avian Seed Dispersal Interactions in a Small Insular Community.” AoB Plants 7, no. 17: 868–872. 10.1093/aobpla/plv017. PMC4372831 25750409

[ece372087-bib-0025] Guimarães, P. R. 2020. “The Structure of Ecological Networks Across Levels of Organization.” Annual Review of Ecology, Evolution, and Systematics 51, no. 1: 433–460. 10.1146/annurev-ecolsys-012220-120819.

[ece372087-bib-0026] Hernández‐Dávila, O. A. , J. Laborde , V. J. Sosa , and C. Díaz‐Castelazo . 2022. “Interaction Network Between Frugivorous Birds and Zoochorous Plants in Cloud Forest Riparian Strips Immersed in Anthropic Landscapes.” Avian Research 13, no. 3: 100046. 10.1016/j.avrs.2022.100046.

[ece372087-bib-0027] Howes, B. , M. González‐suárez , H. J. Jensen , et al. 2023. “Deforestation Alters Species Interactions.” Natural Sciences 3: e20220027. 10.1002/ntls.20220027.

[ece372087-bib-0028] Hsieh, T. C. , K. H. Ma , and A. Chao . 2016. “iNEXT: An R Package for Rarefaction and Extrapolation of Species Diversity (Hill Numbers).” Methods in Ecology and Evolution 7, no. 12: 1451–1456.

[ece372087-bib-0029] Jordano, P. 2016. “Sampling Networks of Ecological Interactions.” Functional Ecology 30: 1883–1893. 10.1111/1365-2435.12763.

[ece372087-bib-0030] Jordano, P. , P. M. Forget , J. E. Lambert , K. Böhning‐Gaese , A. Traveset , and S. J. Wright . 2011. “Frugivores and Seed Dispersal: Mechanisms and Consequences for Biodiversity of a Key Ecological Interaction.” Biology Letters 7: 321–323. 10.1098/rsbl.2010.0986. 21084336 PMC3097856

[ece372087-bib-0031] Li, W. , C. Zhu , I. Grass , et al. 2022. “Plant‐Frugivore Network Simplification Under Habitat Fragmentation Leaves a Small Core of Interacting Generalists.” Communications Biology 5: 1214. 10.1038/s42003-022-04198-8. 36357489 PMC9649668

[ece372087-bib-0032] Liu, F. , Y. Li , K. Zhang , J. Liang , D. Nong , and Z. Huang . 2022. “Habitat Use of the White‐Headed Langurs in Limestone Forest of Southwest Guangxi, China: Seasonality and Group Size Effects.” Ecology and Evolution 12: e9068. 10.1002/ece3.9068. 35813914 PMC9251885

[ece372087-bib-0033] Lu, S. , T. Chen , Z. Huang , Y. Li , and C. Lu . 2021. “Interannual Variation in Food Choice of White‐Headed Langur Inhabiting Limestone Forests in Fusui, Southwest Guangxi, China.” Ecology and Evolution 11, no. 1: 9349–9360. 10.1002/ece3.7726. 34306626 PMC8293718

[ece372087-bib-0034] Mackinnon, J. , and K. Phillipps . 2000. A Field Guide to the Birds of China. Hunan Education Press.

[ece372087-bib-0035] Maruyama, P. K. , C. Melo , C. Pascoal , et al. 2019. “What Is on the Menu for Frugivorous Birds in the Cerrado? Fruiting Phenology and Nutritional Traits Highlight the Importance of Habitat Complementarity.” Acta Botanica Brasilica 33, no. 3: 572–583. 10.1590/0102-33062019abb0221.

[ece372087-bib-0036] Memmott, J. , N. M. Waser , and M. V. Price . 2004. “Tolerance of Pollination Networks to Species Extinctions.” Proceedings of the Royal Society of London. Series B, Biological Sciences 271: 2605–2611. 10.1098/rspb.2004.2909. PMC1691904 15615687

[ece372087-bib-0037] Montoya‐Arango, S. , J. F. Acevedo‐Quintero , and J. L. Parra . 2019. “Abundance and Size of Birds Determine the Position of the Species in Plant‐Frugivore Interaction Networks in Fragmented Forests.” Community Ecology 20, no. 1: 75–82. 10.1556/168.2019.20.1.8.

[ece372087-bib-0038] Moulatlet, G. M. , W. Dáttilo , and F. Villalobos . 2023. “Species‐Level Drivers of Avian Centrality Within Seed‐Dispersal Networks Across Different Levels of Organization.” Journal of Animal Ecology 92, no. 11: 2126–2137. 10.1111/1365-2656.13986. 37454385

[ece372087-bib-0039] Nie, Y. , H. Chen , K. Wang , and Y. Ding . 2014. “Rooting Characteristics of Two Widely Distributed Woody Plant Species Growing in Different Karst Habitats of Southwest China.” Plant Ecology 215: 1099–1109. 10.1007/s11258-014-0369-0.

[ece372087-bib-0040] Ordano, M. , P. G. Blendinger , S. B. Lomáscolo , et al. 2017. “The Role of Trait Combination in the Conspicuousness of Fruit Display Among Bird‐Dispersed Plants.” Functional Ecology 31: 1718–1727. 10.1111/1365-2435.12899.

[ece372087-bib-0041] Pigot, A. L. , T. Bregman , C. Sheard , B. Daly , R. S. Etienne , and J. A. Tobias . 2016. “Quantifying Species Contributions to Ecosystem Processes: A Global Assessment of Functional Trait and Phylogenetic Metrics Across Avian Seed‐Dispersal Networks.” Proceedings of the Royal Society B: Biological Sciences 283: 20161597. 10.1098/rspb.2016.1597. PMC5204145 27928035

[ece372087-bib-0042] Pinheiro, R. B. P. , G. M. F. Felix , and T. M. Lewinsohn . 2022. “Hierarchical Compound Topology Uncovers Complex Structure of Species Interaction Networks.” Journal of Animal Ecology 91, no. 11: 2248–2260. 10.1111/1365-2656.13806. 36054553

[ece372087-bib-0043] Pinto, M. Í. , C. Emer , E. Cazetta , and J. C. Morante‐Filho . 2021. “Deforestation Simplifies Understory Bird Seed‐Dispersal Networks in Human‐Modified Landscapes.” Frontiers in Ecology and Evolution 9: 640210. 10.3389/fevo.2021.640210.

[ece372087-bib-0044] Pizo, M. A. , J. M. Morales , O. Ovaskainen , and T. A. Carlo . 2021. “Frugivory Specialization in Birds and Fruit Chemistry Structure Mutualistic Networks Across the Neotropics.” American Naturalist 197, no. 2: 236–249. 10.1086/712381. 33523785

[ece372087-bib-0045] Quintero, E. , M. A. Pizo , and P. Jordano . 2020. “Fruit Resource Provisioning for Avian Frugivores: The Overlooked Side of Effectiveness in Seed Dispersal Mutualisms.” Journal of Ecology 108, no. 4: 1358–1372. 10.1111/1365-2745.13352.

[ece372087-bib-0046] Quitián, M. , V. Santillán , C. I. Espinosa , et al. 2018. “Elevation‐Dependent Effects of Forest Fragmentation on Plant‐Bird Interaction Networks in the Tropical Andes.” Ecography 41, no. 9: 1497–1506. 10.1111/ecog.03247.

[ece372087-bib-0047] R Core Team . 2024. “R: A Language and Environment for Statistical Computing.” In R Foundation for Statistical Computing. http://www.R‐project.org/.

[ece372087-bib-0048] Ramos‐Robles, M. , E. Andresen , and C. Díaz‐Castelazo . 2016. “Temporal Changes in the Structure of a Plant‐Frugivore Network Are Influenced by Bird Migration and Fruit Availability.” PeerJ 4: e2048. 10.7717/peerj.2048. 27330852 PMC4906665

[ece372087-bib-0049] Ruggera, R. A. , P. G. Blendinger , M. D. Gomez , and C. Marshak . 2016. “Linking Structure and Functionality in Mutualistic Networks: Do Core Frugivores Disperse More Seeds Than Peripheral Species?” Oikos 125: 541–555. 10.1111/oik.02204.

[ece372087-bib-0050] Rumeu, B. , J. P. González‐Varo , C. Castro , et al. 2023. “Increasing Efficiency and Reducing Bias in the Sampling of Seed‐Dispersal Interactions Based on Mist‐Netted Birds.” Oikos 2: e09261. 10.1111/oik.09261.

[ece372087-bib-0051] Saavedra, F. , I. Hensen , S. G. Beck , et al. 2014. “Functional Importance of Avian Seed Dispersers Changes in Response to Human‐Induced Forest Edges in Tropical Seed‐Dispersal Networks.” Oecologia 176: 837–848. 10.1007/s00442-014-3056-x. 25182931

[ece372087-bib-0052] Saavedra, S. , D. B. Stouffer , B. Uzzi , and J. Bascompte . 2011. “Strong Contributors to Network Persistence Are the Most Vulnerable to Extinction.” Nature 478: 233–235. 10.1038/nature10433. 21918515

[ece372087-bib-0053] Santos, T. D. , and A. S. Ribeiro . 2023. “Mutualistic Interaction Network Structure Between Bird and Plant Species in a Semi‐Arid Neotropical Environment.” Acta Oecologica 118: 103897. 10.1016/j.actao.2023.103897.

[ece372087-bib-0054] Schleuning, M. , N. Blüthgen , M. Flörchinger , J. Braun , H. M. Schaefer , and K. Böhning‐Gaese . 2011. “Specialization and Interaction Strength in a Tropical Plant‐Frugivore Network Differ Among Forest Strata.” Ecology 92, no. 1: 26–36. 10.1890/09-1842.1. 21560673

[ece372087-bib-0055] Schupp, E. W. , P. Jordano , and J. M. Gómez . 2010. “Seed Dispersal Effectiveness Revisited: A Conceptual Review.” New Phytologist 188: 333–353. 10.1111/J.1469-8137.2010.03402.X. 20673283

[ece372087-bib-0056] Sebastián‐González, E. 2017. “Drivers of Species' Role in Avian Seed‐Dispersal Mutualistic Networks.” Journal of Animal Ecology 86: 878–887. 10.1111/1365-2656.12686. 28415137

[ece372087-bib-0057] Sebastián‐González, E. , B. Dalsgaard , B. Sandel , and P. R. Guimarães . 2015. “Macroecological Trends in Nestedness and Modularity of Seed‐Dispersal Networks: Human Impact Matters.” Global Ecology and Biogeography 24: 293–303. 10.1111/geb.12270.

[ece372087-bib-0058] Silva, A. M. , P. K. Maruyama , L. P. M. Paniago , and C. Melo . 2016. “Modularity in Ecological Networks Between Frugivorous Birds and Congeneric Plant Species.” Journal of Tropical Ecology 32: 526–535. 10.1017/S0266467416000444.

[ece372087-bib-0059] Spotswood, E. N. , J. Y. Meyer , and J. W. Bartolome . 2012. “An Invasive Tree Alters the Structure of Seed Dispersal Networks Between Birds and Plants in French Polynesia.” Journal of Biogeography 39, no. 11: 2007–2020. 10.1111/j.1365-2699.2012.02688.

[ece372087-bib-0060] Teodosio‐Faustino, I. A. , E. Chávez‐González , and E. R. Inzunza . 2021. “In a Neotropical Periurban Park, Fruit Consumption by Birds Seems to Be a Random Process.” Frontiers in Ecology and Evolution 9: 630150. 10.3389/fevo.2021.630150.

[ece372087-bib-0061] Tinoco, B. A. , C. H. Graham , J. M. Aguilar , and M. Schleuning . 2017. “Effects of Hummingbird Morphology on Specialization in Pollination Networks Vary With Resource Availability.” Oikos 126: 52–60. 10.1111/oik.02998.

[ece372087-bib-0062] Ulrich, W. , and N. J. Gotelli . 2007. “Null Model Analysis of Species Nestedness Patterns.” Ecology 88: 1824–1831. 10.1890/06-1208.1. 17645028

[ece372087-bib-0063] Vaughan, I. P. , N. J. Gotelli , J. Memmott , C. E. Pearson , G. Woodward , and W. O. C. Symondson . 2018. “Econullnetr: An R Package Using Null Models to Analyse the Structure of Ecological Networks and Identify Resource Selection.” Methods in Ecology and Evolution 9: 728–733. 10.1111/2041-210X.12907.

[ece372087-bib-0064] Villegas, M. , C. Mayta , C. L. López , I. Hensen , and S. C. Gallegos . 2024. “Bird Communities Respond to the Seasonal Fruit and Flower Availability in a Fragmented Tropical Andean Landscape.” Ornitología Neotropical 35, no. 1: 38–45. 10.58843/ornneo.v35i1.1281.

[ece372087-bib-0065] Vizentin‐Bugoni, J. , C. E. Tarwater , J. T. Foster , et al. 2019. “Structure, Spatial Dynamics, and Stability of Novel Seed Dispersal Mutualistic Networks in Hawai'i.” Science 364: 78–82. 10.1126/science.aau8751. 30948550

[ece372087-bib-0066] Wang, G. , Y. Huang , W. Yao , et al. 2023. “Structure and Characteristics of the Plant‐Frugivore Bird Network From the Guilin Botanical Garden.” PeerJ 11: e15028. 10.7717/peerj.15028. 36945357 PMC10024898

[ece372087-bib-0067] Wotton, D. M. , and D. Kelly . 2012. “Do Larger Frugivores Move Seeds Further? Body Size, Seed Dispersal Distance, and a Case Study of a Large, Sedentary Pigeon.” Journal of Biogeography 39, no. 11: 1973–1983. 10.1111/jbi.12000.

[ece372087-bib-0068] Yuan, Q. , and R. Li . 2023. “The Negative Impacts of Human Activities on the Ecological Corridor in the Karst Highly Urbanized Area Are Gradually Diminishing: A Case Study From the Karst Mountain Cities in Southwest China.” Ecological Indicators 157: 111257. 10.1016/j.ecolind.2023.111257.

[ece372087-bib-0069] Zhang, K. , Q. Zhou , H. Xu , and Z. Huang . 2020. “Effect of Group Size on Time Budgets and Ranging Behavior of White‐Headed Langurs in Limestone Forest, Southwest China.” Folia Primatologica 91, no. 3: 188–201. 10.1159/000502812. 31665731

[ece372087-bib-0070] Zhang, M. , C. Lu , Q. Han , and C. Lu . 2022. “Structure and Characteristics of Plant‐Frugivore Network in an Urban Park: A Case Study in Nanjing Botanical Garden Mem. Sun Yat‐Sen.” Diversity 14: 71. 10.3390/d14020071.

[ece372087-bib-0071] Zhao, Z. J. 2001. A Handbook of the Birds of China. Science and Technology Press.

[ece372087-bib-0072] Zhu, C. , B. Dalsgaard , W. Li , et al. 2024. “Generalist and Topologically Central Avian Frugivores Promote Plant Invasion Unequally Across Land‐Bridge Islands.” Ecology 105, no. 2: e4216. 10.1002/ecy.4216. 38037487

