# Supplementary material for: Interaction Network Between Frugivorous Birds and Plants in Karst Habitats
Source: Ecol Evol. 2025 Sep 17;15(9):e72087. doi: 10.1002/ece3.72087 (PMC12443814; doi:10.1002/ece3.72087)
Supplement: Supplementary file 1 — Table S1: Observation time per plant species: Overall, rainy season, and dry season. [file ECE3-15-e72087-s001.docx]

**Table S.  Observation time per plant species: Overall, rainy season, and dry season**

| Plant species | Total observation time/h | Observation time during rainy season/h | Observation time during dry season/h |
| --- | --- | --- | --- |
| *Ficus concinna* | 71.50 | 46.07 | 25.43 |
| *Camphora officinarum* | 49.77 | 37.57 | 12.20 |
| *Ficus altissima* | 37.45 | 29.78 | 7.67 |
| *Melia azedarach* | 28.98 | 0.00 | 28.98 |
| *Flueggea virosa* | 27.97 | 17.00 | 10.97 |
| *Bischofia javanica* | 23.85 | 0.00 | 23.85 |
| *Phyllanthus reticulatus* | 17.78 | 3.80 | 13.98 |
| *Ficus tinctoria* | 8.53 | 8.53 | 0.00 |
| *Broussonetia papyrifera* | 9.27 | 9.27 | 0.00 |
| *Causonis japonica* | 10.22 | 1.10 | 9.12 |
| *Maclura tricuspidata* | 9.27 | 0.00 | 9.27 |
| *Syzygium cumini* | 6.03 | 6.03 | 0.00 |
| *Persicaria chinensis* | 7.22 | 0.00 | 7.22 |
| *Sageretia thea* | 3.37 | 0.00 | 3.37 |
| Total | 311.21 | 159.15 | 152.06 |
